# Prevalence and associated factors of ADHD symptoms among higher education students in Southern Ethiopia

**DOI:** 10.3389/fpsyt.2025.1566847

**Published:** 2026-01-14

**Authors:** Chalachew Kassaw, Aleksandra Meshcheriakova, Biazin Yenealem Mekuriaw, Valeriia Demareva

**Affiliations:** 1 Department of Psychiatry, Dilla University, Dilla, Ethiopia; 2 Department of Cyber Psychology, Lobachevsky University, Nizhniy Novgorod, Russia

**Keywords:** ADHD symptoms, Dilla University, Ethiopia, higher education students, risk factors

## Abstract

**Introduction:**

Adult Attention-Deficit/Hyperactivity Disorder (ADHD), a neurodevelopmental condition originating in childhood, manifests as persistent inattention and/or hyperactivity-impulsivity, affecting occupational and daily functioning. This study examined the prevalence and associated factors of ADHD symptoms among higher education students in southern Ethiopia, aiming to elucidate the challenges faced by this population and inform targeted support strategies.

**Methodology:**

This cross-sectional study was conducted from November to December 2024 among undergraduate students at Dilla University. ADHD symptom severity was assessed using the self-reported Adult ADHD Self-Report Scale (ASRS-v1.1). A random sampling technique was employed to select participants, and a self-administered questionnaire was used to collect data. Descriptive statistics were used to summarize the results, and multivariate logistic regression analysis was performed to identify variables associated with ADHD symptoms.

**Results:**

A total of 513 respondents participated in the current study. Of all respondents, 218 (42%) were female. The mean grade point average (GPA) of respondents was 3.43, and 218 (42%) scored above this average. The prevalence of High ADHD symptoms among these higher education students was 25.1% (95% CI: 22.1%–27.7%). Compared to students with low ADHD symptoms, students with High symptoms were more likely to live in rural areas, have a current grade point average (GPA)< 3.43, study for less than four hours during exam periods, have a history of childhood infections, experience greater test anxiety, show poor cognitive function scores, and engage in high social media use.

**Conclusions:**

This study revealed that one in four students experienced High ADHD symptoms, and identified some of the associated potentially modifiable factors among university students attending Dilla University, southern Ethiopia. These findings suggest the need to screen for ADHD symptoms and explore their role in the academic success of university students. Interventions should address psychosocial and cognitive aspects of academic outcomes in higher education students in low-resource settings. Moreover, multicenter and interventional studies with larger samples are warranted to validate these results and optimize intervention strategies.

## Introduction

Attention-Deficit/Hyperactivity Disorder (ADHD) is a neurodevelopmental condition characterized by persistent patterns of inattention, hyperactivity, and impulsivity. The onset typically begins in childhood and continues into adulthood. It significantly affects various aspects of life and academic functioning (1, 2). The core behavioral symptoms of ADHD are frequently linked to cognitive difficulties, particularly in attentional control, processing speed, and executive function, such as working memory (3).

### Burden of ADHD symptom in higher education settings

The academic environment brings unique and intensive challenges to students with elevated symptoms of Attention-Deficit/Hyperactivity Disorder (ADHD), resulting in a significant and often underestimated burden within higher education settings. The global estimate of ADHD symptoms in higher education settings was 15.9% (4) Attempts to quantify the magnitude of ADHD symptoms among higher education students have been reported across different parts of the world. The prevalence of elevated ADHD symptoms among Mid-Atlantic regions of the United States using DSM-5 criteria and cohort method was 15.7% (5) and 7% in universities in the United Kingdom using the Conners’ Adult ADHD Self-Rating Scale (6). The magnitude of above threshold ADHD symptoms using the World Health Organization (WHO) 18-item Adult ADHD Self-Report Scale v 1.1 Symptom Checklist was 10.1% among Chinese medical students (7), 5.7% among Korean university students (8), and 34.8% in Pakistani medical students (9). Another study revealed the magnitude of high-level ADHD symptoms using the DSM IV-Based Diagnostic Screening and Rating Scale in Turkey was 10.1% (10), and 25% in Thai medical students (11) and 16.5% among Iranian university students (12). High ADHD symptoms were seen in 8.3% of students in Belgium (as measured using ASRS-6) (13) and 36.8% of students in Brazil using the ASRS-18 (14).

In Africa, the magnitude of ADHD symptoms using the 18-item Adult ADHD Self-Report Scale v 1.1 Symptom Checklist was 23.7% at Eldoret University in Kenya (15), 24.4% in Cameroon (16), and 20% at Gondar University in Ethiopia using six-item Adult Self-Report Scale-V1.1 (ASRS-V1.1) (17). The previous literature is predominantly from Western settings and middle-income countries, which hinders the global understanding of ADHD symptom in higher education settings. Although we recognize that psychometric screening instruments do not possess the diagnostic equivalence of a formal clinical evaluation, the resulting prevalence data on elevated symptom burden nonetheless constitute a robust and critical metric for informing population-level mental health planning. There is a need to examine how the academic and clinical presentation of ADHD is perceived within diverse socio-cultural contexts, such as that of Ethiopia, which informs the focus of the subsequent discussion.

### Cultural understanding of ADHD symptom manifestation in Ethiopian context

The symptoms of inattention and hyperactivity, such as daydreaming, high energy, and curiosity, may be considered as spiritual possession rather than psychological issues (18). These explanations might lead to underreporting of ADHD symptoms and misinterpretation of behaviors. Furthermore, the symptoms of inattentiveness may be masked by a student’s social network. Together, these factors may lead to underestimating the burden of ADHD in this population. Conversely, the sociocultural context may be associated with more severe behaviors.

The higher educational system in Ethiopia usually depends on routine memorization and large class sizes, which might not be appropriate for the learning styles of students with ADHD symptoms. The symptoms of inattention and hyperactivity may be exacerbated due to the need to sit still and listen for extended periods (19).

Last, hyperactivity may cause secondary anxiety and psychological distress due to the violation of social norms and family expectations. Adults with this condition often visit religious and traditional healers to obtain relief due to the cultural attitudes attached to it (20). In light of these issues, a better understanding of ADHD, its risk factors, and outcomes for college students, within the Ethiopian context, is needed.

### Associated factors with ADHD symptom

Identifying modifiable environmental, psychological, and behavioral factors is important for organizing targeted, context-specific early intervention and support programs within the higher education setting. The theoretical framework for this study was based on Sonuga-Barke and Halperin’s developmental model of ADHD, which conceptualizes the disorder’s severity and persistence as resulting from dynamic interactions between biological risk and contextual/psychosocial factors across the lifespan. Low socioeconomic status, perinatal stressors, genetic predisposition, childhood adversity, and authoritarian parenting approaches have all been associated with more severe ADHD symptom expression (21–25). Strong associations have been observed between parental chronic health conditions and mental illness with the occurrence of ADHD symptoms in children (26).

Repeated bacterial and viral infections during childhood have been highly associated with ADHD symptoms during later adulthood (27).

### Impairing outcomes of elevated ADHD symptoms

The impairing outcomes were based on a framework primarily drawn from the neurodevelopmental model of executive dysfunction and the ecological systems theory that reflects the impact of ADHD symptoms on academic functioning, mental health comorbidity, self-efficacy, and financial and vocational work difficulties (28).

Attentional deficits significantly impair lecture engagement, assignment completion, and knowledge acquisition (29). Students with ADHD symptoms have difficulty in controlling their thoughts and behavior that directly affect self-awareness, learning regulation, and utilizing effective study strategies (30–34). Students with ADHD also have lower GPAs, difficulty engaging in procedural academic tasks or long study hours, are more likely to have incomplete assignments, worry about their grades, and are at increased risk of academic failure (34–39). The academic struggles of students with ADHD can interact with their social and emotional difficulties, increasing the need for psychological health services (40, 41).

Adults with ADHD symptoms often experience difficulty with self-esteem, getting adequate sleep, and are more likely to engage in problematic substance use (42). Furthermore, many individuals with ADHD symptoms have difficulty with impulsivity and reward processing, which can increase the risk of depression and psychological distress (43).

### Aim and significance of the study

Understanding the burden of ADHD symptoms in higher education students is crucial to designing interventions that enhance students’ lecture engagement, assignment completion, and knowledge acquisition (44, 45). However, there are limited studies examining the prevalence of elevated ADHD symptoms in Ethiopian higher education institutions, and no study in the Southern part of the country. Thus, the current study aimed to determine: (i) the prevalence of ADHD symptoms among higher education students in Southern Ethiopia; and (ii) associated factors and impairing outcomes of High ADHD symptoms, including aspects of cognitive functioning, academic achievement, psychosocial health, and various socio-demographic factors. This knowledge may help to design targeted interventions to prevent or reduce the impact of associated risk factors, as well as minimize impairment to students’ personal, social, and academic functioning. Assessing the magnitude of the relations between ADHD symptoms and associated factors would contribute to a more comprehensive understanding of the factors associated with High ADHD symptoms in this specific cultural and societal context. Understanding the impairing outcome of High ADHD symptoms related to academic outcomes in the Southern Ethiopian university setting is essential for developing targeted support and interventions. Furthermore, the findings would contribute to a better understanding of the manifestation and correlates of ADHD symptoms in a developing country context and can inform the development of culturally sensitive interventions and support services.

## Materials and methods

### Study area and period

The study was conducted at Dilla University, located in the Gedeo Zone within the Dilla City Administration of the Southern Nations, Nationalities, and Peoples’ Region (SNNPR), Ethiopia. The university is situated approximately 365 kilometers from the capital city, Addis Ababa. Data collection took place across the university’s three campuses: the main campus, Odaya campus, and the Medicine and Health campus. Agriculture, Science, Technology, Engineering, and Mathematics (STEM), social science, and health science. The university has Bachelor’s, Master’s, and Doctor of Philosophy academic programs.

### Sample size determination

The sample size was calculated using the single population proportion formula, based on the proportion of ADHD symptoms reported among undergraduate Gondar university students (20%) (17).The formula used was:


$$n=\frac{Z_{\alpha /2}^{2}*p*q}{d^{2}}=246$$

where:

$Z_{\alpha /2}^{2}$=1.96 (for a 95% confidence level)

p =0.2 (estimated prevalence of ADHD symptoms)

q =0.8 ( 1−p)

d =0.05 (margin of error)

With a 5% non-response rate, the adjusted sample size becomes:


n=246+24=270

Given the use of multistage sampling, the sample size was doubled to account for potential stratification and increased variability, resulting in a final sample size of 540.

### Sampling procedure

**Figure 1:**
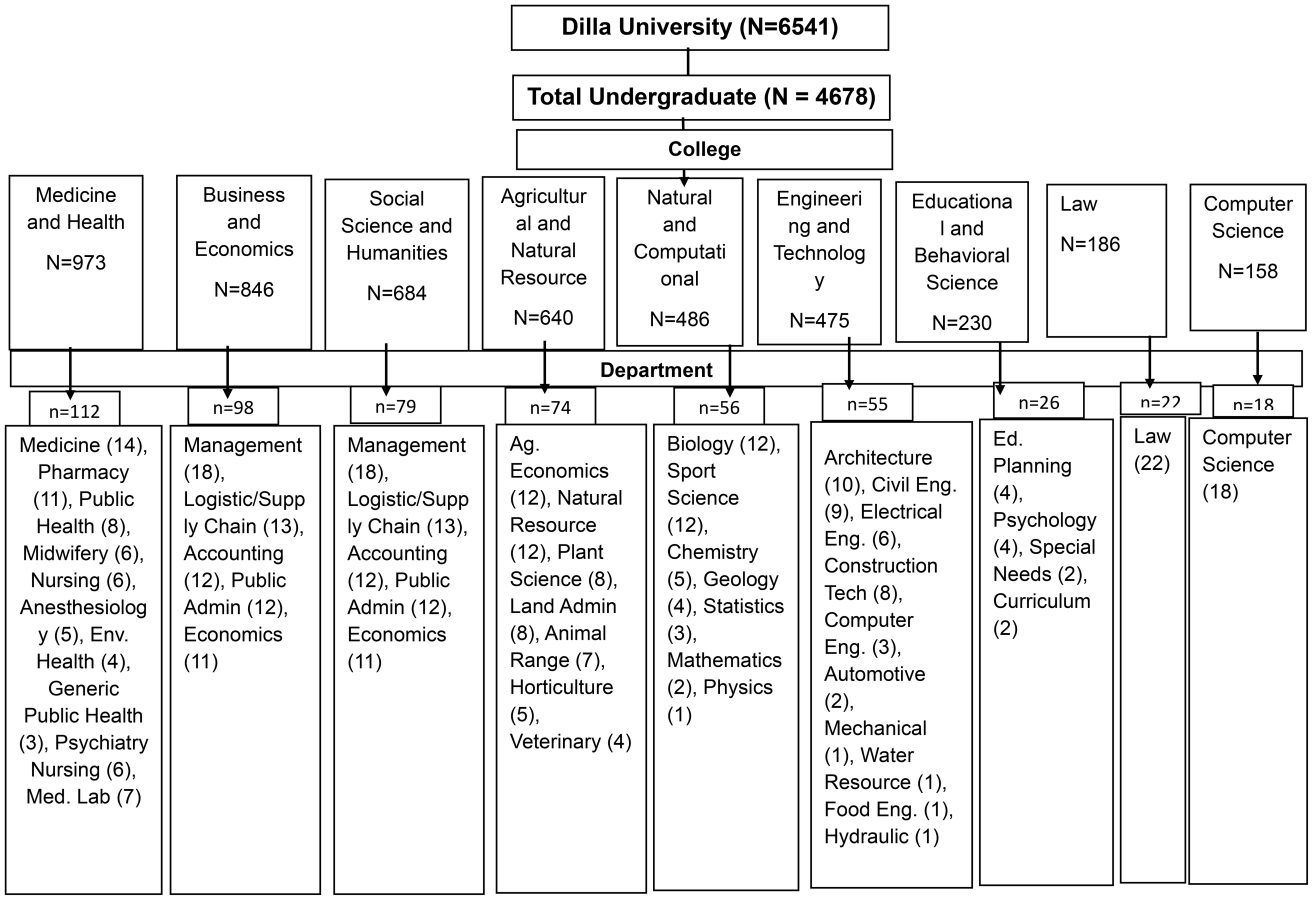
Schematic representation of sample selection based on proportional method (n=540).

The study targeted actively registered undergraduate students at Dilla University. The study included a diverse range of academic disciplines to ensure a representative sample of the student population. The study employed a multi-stage simple random sampling technique. First, we obtained a list of all academic disciplines in Dilla University. From this list, a simple random sample of 9 academic schools was selected. Subsequently, within each selected academic school, we obtained a list of all enrolled students from the registrar’s office. Using a computer-generated random number table, we then selected a simple random sample of students from each department proportional to the department’s size within the institution. To ensure a representative sample from each college and department, the researchers employed a simple random sampling (SRS) technique (46). Additionally, proportional allocation was applied across the different colleges, departments, and academic years to ensure that the sample accurately reflected the university’s population distribution (**Figure 1**).

### Eligibility criteria

The eligibility of students to participate in the current study was full-time students admitted through the national, centralized higher education entrance system, excluding private, extension, or evening program students. The exclusion criteria included students with acute mental or physical difficulties that required immediate medical or psychological attention and hindered their ability to give information for the current study.

### Measures

Socio-demographics, clinical history, and all scale scores, were collected via self-report. Sociodemographic characteristics included age, gender, residence, parents’ educational status, stable parent, and monthly pocket money. Academic factors included scientific area, department, year of study, number of students in the class, average study hours, and worrying about academic performance.

Family history of mental illnesses was defined as respondents reporting that a family member had been diagnosed with a psychiatric disorder, coded no or yes.

History of childhood infection was defined as respondents reporting that they had experienced a severe childhood infection requiring hospitalization, as they heard from their parents, and was coded no or yes (27).

Lifetime family history was operationalized as a non-diagnostic, self-reported measure indicating whether the respondent reported any lifetime use of any substance (e.g., alcohol, nicotine, illicit drugs) by at least one parent.

Stable parent reflected whether the respondents reported they had both mother and father in home, categorized as “stable parent.” Separated/only mother or father alive was categorized as “un-stable parent”.

### ADHD symptoms

The Adult Self-Report Scale (ASRS V1.1) was employed to assess ADHD symptoms. This 18-item self-report instrument utilizes a 5-point Likert scale (Never, Rarely, Sometimes, Often, Very Often) to measure the frequency of inattention (9 items) and hyperactivity/impulsivity (9 items) symptoms over the preceding 6 months. Each item is scored from 0 to 4, yielding a total symptom score ranging from 0 to 72. A cutoff score of >30 was used in this study to indicate the presence of ADHD symptoms (47). The tool has strong concurrent validity and discriminant validity by showing a high correlation with established, semi-structured clinical interviews for ADHD and distinguishes ADHD symptoms from symptoms of other conditions. The tool is a valid measure with a clear and well-defined two-factor model fit structure for confirmatory analysis that aligns with the established clinical understanding of ADHD. The ASRS V1.1 has demonstrated high internal consistency in previous research, with reported Cronbach’s alpha coefficients of 0.88 (48, 49). In the current study, Cronbach’s alpha value was 0.92.

### Cognitive function

The Mizan Meta-Memory and Meta-Concentration Scale (MMSS) assesses two distinct aspects of metacognition: meta-memory (comprising 5 items) and meta-concentration (comprising 4 items). Participants respond to each item using a 5-point Likert scale, with response options ranging from 1 (“strongly disagree”) to 5 (“strongly agree”). The total score on the MMSS can range from 9 to 45, where higher scores indicate greater perceived metacognitive ability. The Internal Consistency of the tool in a previous study done in Nigeria showed Cronbach’s alpha values of 0.875 for the MMSS, 0.808 for the meta-memory subscale, and 0.857 for the meta-concentration subscale. The MMSS appears to have adequate construct validity, as evidenced by Exploratory factor analysis (EFA) and confirmatory factor analysis (CFA) (50). Another study done in Ethiopia reported good internal consistency, with Cronbach’s alpha at 0.84 for meta-memory and 0.80 for meta-concentration (51). In the current study, Cronbach’s alpha value for the meta-memory subscale was 0.82, and 0.76 for the meta-concentration subscale.

The Mizan meta-memory and meta-concentration scale

for students (MMSS)

**Mizan Meta-Memory and Meta-Concentration Scale for Students (MMSS)** The total score ranges from 9 to 45, with higher scores indicating better metacognitive ability (52). Mean scores were used to categorize students into groups with good or poor metacognitive ability (53). The mean score of MMSS in this study was 23/45. In the current study, the tool’s Cronbach’s alpha value was 0.86.

### Academic achievement (Grade Point Average)

GPA reflects overall academic performance, calculated as a weighted average of students’ grades (54). In this study, participants with a GPA below the mean were classified as poor academic achievers, and those with a GPA above the mean as good academic achievers. Students self-disclosed their GPA, and the authors checked its validity with the registrar’s office.

### Social support

The Oslo Social Support Scale (OSSS-3) is a brief, three-item instrument designed to assess perceived social support (55). This study utilized the OSSS-3 to measure participants’ levels of social support. It was a Likert scale, and responses were scored from 1 to 5 for each of the three questions. The scale yields a total score ranging from 3 to 14, where lower scores indicate poorer perceived social support and higher scores reflect stronger support. Previous research has demonstrated acceptable psychometric properties for the OSSS-3, including good internal consistency and construct validity across various populations (56). The OSSS-3 is commonly used in social science and health research to examine the relationship between social support and a range of outcome variables, such as mental health, well-being, and physical health indicators (57).

The scale categorizes individuals into groups with poor (3–8), moderate (9–11), or strong (12–14) social support (55). The scale has satisfactory internal consistency, with a Cronbach’s alpha of 0.64 in a previous study done in Addis Ababa University (58). In the current study, Cronbach’s alpha value was 0.84.

### Sleep quality

The Pittsburgh Sleep Quality Index (PSQI) is a 19-item self-report measure assessing sleep quality over the past month. It is a widely utilized and validated questionnaire designed to evaluate various aspects of sleep and its impact on daily functioning. The average internal consistency or Cronbach’s alpha coefficient of the tool was 0.72 (59). The PSQI has shown moderate-to-good test-retest reliability over varying time intervals (e.g., one week to one month), indicating that the scores are relatively stable over time in the absence of significant changes in sleep patterns (60). The seven components (subjective sleep quality, sleep latency, sleep duration, habitual sleep efficiency, sleep disturbances, sleeping medication, and daytime dysfunction) of the PSQI align with theoretical constructs of sleep quality. Factor analytic studies have generally supported the multidimensional nature of the PSQI, although the exact factor structure can sometimes vary slightly across different populations (61). The global PSQI score greater than 5 has been established as a reliable cutoff point for distinguishing between “good” and “poor” sleepers, demonstrating a sensitivity of 82% and a specificity of 56.2% in the original validation study (62). The PSQI has demonstrated its ability to differentiate between individuals with and without various sleep disorders, as well as between individuals with and without certain medical and psychiatric conditions known to affect sleep (e.g., insomnia, depression, chronic pain) (63). The PSQI includes seven components measuring subjective sleep quality, sleep latency, sleep duration, efficiency, disturbances, medication use, and daytime dysfunction, generating a global score ranging from 0 to 21 (64). A score >5/21 indicates poor sleep, with 82% sensitivity and 56.2% specificity. In the current study, Cronbach’s alpha value was 0.87.

### Perceived stress

The Perceived Stress Scale (PSS-10), a widely used instrument for assessing the degree to which situations in one’s life are appraised as stressful, has demonstrated acceptable psychometric properties in various populations, including evidence of internal consistency and construct validity. Specifically, studies conducted on the Amharic and Afan Oromo versions of the PSS-10 among Ethiopian higher education students have reported Cronbach’s alpha coefficients ranging from 0.75 to 0.85, indicating good internal reliability (65). Within the Ethiopian higher education context, the PSS-10 has been employed to assess perceived stress levels about academic performance, substance use, psychological distress (such as anxiety and depression), and coping mechanisms. The scale has 10 items designed to assess the subjective experience of stress, focusing on the unpredictability, uncontrollability, and overload aspects of life events over the past month. The response format for each question is a 5-point Likert scale ranging from 0 (Never) to 4 (Very Often). In Ethiopian higher education students, a score ≥13/40 indicates significant perceived stress (66, 67). In the current study, the tool’s Cronbach’s alpha value was 0.80.

### Social media addiction

The Bergen Social Media Addiction Scale (BSMAS), a 6-item self-report instrument designed to assess social media addiction severity over the past year, demonstrates robust psychometric properties. It’s reported that Cronbach’s alpha of 0.91 indicates strong internal consistency, suggesting that the items reliably measure the same underlying construct validity (68). The scale has a 5-point Likert response scale (1 = Very rarely, 2 = Rarely, 3 = Sometimes, 4 = Often, and 5 = Very often), with scores ranging from 6to 30, with higher scores suggesting problematic social media usage. Previous studies employing the BSMAS have often examined its relationship with various outcome variables, such as measures of mental health (e.g., anxiety, depression), sleep quality, academic performance, and interpersonal relationships, providing insights into the potential real-world consequences associated with social media addiction (69). The mean score was used to classify students into high or low social media addiction categories (70).

In the current study, the mean score of social media use was 17/30. In the current study, the tool’s Cronbach’s alpha value was 0.78.

### Depression

The Beck Depression Inventory (BDI-II), a widely used 21-item self-report questionnaire, possesses well-established psychometric properties for measuring the severity of depressive symptoms. Its reliability and validity have been demonstrated across numerous studies and populations (71).

In previous use, the BDI has served as a critical tool in both clinical settings for diagnosing and monitoring depression and in research to investigate the prevalence and correlates of depressive symptoms across various demographic groups and conditions (72). Regarding its internal consistency, meta-analyses have reported Cronbach’s alpha coefficients ranging from 0.81 to 0.93 across various populations, indicating high internal consistency (73). The test-retest reliability of the BDI, assessing the stability of scores over time, has shown good to excellent coefficients, with reported values (Pearson’s) ranging from 0.73 to 0.96 over various time intervals (74). The Exploratory Factor Analysis (EFA) and Confirmatory Factor Analysis (CFA) values of the tool are acceptable in a higher education setting. Respondents rate the intensity of each of 21 symptoms on a 4-point scale ranging from 0 to 3. The sum of these ratings yields a total score ranging from 0 to 63, providing a quantitative measure of depression severity. This total score is often categorized into levels such as minimal (0–13), mild (14–19), moderate (20–28), and severe (29–63) (75). Students with a score ≤ 13/63 were classified as “No depression,” while those with scores above 13/63 were classified as “Depressed” (76). In the current study, the tool’s Cronbach’s alpha value was 0.87.

### Substance use

The Alcohol, Smoking, and Substance Involvement Screening Test (ASSIST-3.0) was used to assess lifetime and current use of substances such as alcohol, tobacco, chewing gum, and cannabis. If the respondents reported that they used any of the substances over the past 3 months, it was categorized as ‘Yes’ for current substance, and if not, categorized as ‘No’. The content validity, concurrent validity, and factor structure, as examined through exploratory and confirmatory factor analyses, showed acceptable use of the assessment tool in a higher education setting. The scale has a sensitivity of 97% and a specificity of 90% (77, 78). In the current study, the tool’s Cronbach’s alpha value was 0.86.

### Coping strategy

The Coping Inventory for Stressful Situations-21 (CISS-21), a 21-item self-report instrument, is designed to measure three distinct coping styles: task-oriented coping (focusing on problem-solving), emotion-oriented coping (focusing on managing emotional responses), and avoidance-oriented coping (focusing on avoiding the stressful situation) (79). In terms of psychometric properties, the CISS-21 has demonstrated acceptable internal consistency, with reported Cronbach’s alpha coefficients generally exceeding 0.70 for its subscales, suggesting that the items within each coping style consistently measure the intended construct. The scale’s design and composition comprise three 7-item subscales, each corresponding to one of the three coping styles. Each item is rated on a 5-point Likert scale (typically ranging from 1 “Not at all” to 5 “Very much”), with higher scores on a subscale indicating a stronger tendency to utilize that particular coping style (80). The exploratory and confirmatory factor analysis, content validity, and concurrent validity showed an acceptable assessment tool in a higher education setting. For analytical purposes, the coping styles have often been categorized as “high” or “low” based on the mean score of the respective subscale within a given sample. In the current study, the mean scores for avoidance and emotional coping were ≤ 16/35, and task coping was ≤ 18/35. The CISS-21 has been widely employed across diverse populations and stressful contexts, including academic stress, health-related challenges, and occupational stress, to understand individuals’ preferred ways of responding to adversity (81, 82). In the current study, the tool’s Cronbach’s alpha value was 0.89.

### Self-esteem

The Rosenberg Self-Esteem Scale (RSE), a widely utilized instrument for assessing global self-esteem levels, demonstrates sound psychometric properties. It exhibits an internal consistency (Cronbach’s alpha) of 0.77, indicating acceptable reliability among its items. Furthermore, within Ethiopian populations, the scale has shown high sensitivity (87%) and specificity (79%) in identifying individuals with low self-esteem (83). Its scale design and composition comprise 10 items, each rated on a 4-point Likert scale ranging from “Strongly agree” to “Strongly disagree.” Total scores range from 0 to 30, with a score below ≤ 15 typically indicating low self-esteem (84).

The scale has been extensively employed across diverse cultural contexts and age groups to investigate self-esteem about a multitude of outcome variables. The exploratory and confirmatory factor analysis, content validity, and concurrent validity supported the use of this assessment tool in a resource higher education setting. These include mental health indicators such as depression and anxiety, social adjustment, academic achievement, and overall well-being (85). In the current study, the tool Cronbach’s alpha value was 0.88.

### Educational environment

The Dundee Ready Education Environment Measure (DREEM) is a 50-item survey used to assess students’ perceptions of the educational environment in higher education. It includes five subscales: Academic Self-Perception, Student Perception of Teachers, Student’s Social Perception, Student Perceptions of Atmosphere, and Student Perceptions of Learning. The total score ranges from 0 to 200, with higher scores indicating a more positive environment. Scores between 101–150 suggest a more positive than negative environment, while scores of 150–200 indicate an excellent learning experience (86). The survey measures student perceptions of teachers (0–44), academic self-perception (0–32), and student social perception (0–28) using a Likert scale with descriptive anchors for each range (87). Regarding its internal consistency, studies have reported Cronbach’s alpha coefficients for the overall DREEM scale ranging from 0.82 to 0.95, indicating high internal consistency (88). The internal consistency of the five subscales (Students’ Perception of Learning, Students’ Perception of Teachers, Students’ Academic Self-Perception, Students’ Perception of Atmosphere, and Students’ Social Self-Perception) has shown more variability, with some studies reporting values below 0.70 for certain subscales. Scores above the mean were categorized as “high,” while scores below the mean were categorized as “low” (89). The exploratory and confirmatory factor analysis, content validity, and concurrent validity showed an acceptable assessment tool for use in low-resource higher education settings. The test-retest reliability of the DREEM, assessing the stability of scores over time, has been reported as moderate, with correlation coefficients ranging from 0.59 to 0.71 over different time intervals (90). In the current study, the tool’s Cronbach’s alpha value was 0.84.

### Data collection procedures

English is the official language of instruction at Dilla University for higher education students. Data collection was conducted using a self-administered English version of a questionnaire that gathered information on socio-demographic characteristics, academic factors (e.g., GPA, academic self-perception), psychosocial factors (e.g., social media use, depression, sleep quality, perceived stress, self-esteem, social support), and health-related factors (e.g., chronic illness).

Data for this study were collected using pencil-and-paper questionnaires. Participants read and answered the questions at their own pace and in their own time. Anonymity and confidentiality were emphasized to encourage honest responses. On average, it took approximately 30–50 minutes to complete all of the measures included in the questionnaires. This timeframe included providing informed consent, completing the demographic information section, and responding to the ADHD symptom scales and associated factor questions. Data collectors were trained to emphasize that there was no time limit and participants could have taken as much time as needed.

### Data quality control

To ensure high-quality data, the study implemented rigorous quality control measures, including careful questionnaire design, training for data collectors, a pre-test, and close supervisory monitoring throughout data collection. Before data collection, all data collectors underwent a comprehensive two-day training program. This training covered the following aspects: Study objectives and procedures, Ethical considerations, administering the questionnaire, handling participant queries, and Data quality assurance. Following the training, a pre-test was conducted on a sample of 54 students who were not included in the final study sample. The pre-test aimed to assess the clarity and comprehensibility of the questionnaire items, evaluate the time taken for completion, identify any potential challenges or ambiguities in the data collection process, and evaluate the performance of the data collectors. Based on the feedback received during the pre-test, minor revisions were made to the questionnaire wording and the data collection protocol to enhance clarity and efficiency. Participants were not directly compensated financially for their participation in this study. However, we ensured that their participation was voluntary and that they were fully informed about the study’s purpose and their rights as participants. We expressed our sincere gratitude for their time and contribution to this important research. We believe that the potential benefits of this research in understanding and addressing ADHD symptoms among higher education students in Southern Ethiopia outweigh the lack of direct compensation.

### Data processing and analysis

The data was entered into Epi-Data 7 software and analyzed using SPSS version 25. Binary and multivariate logistic regression analysis was employed to examine the relationship between ADHD symptoms and several independent risk factors and outcomes. The cut score >30/72 was used to identify the presence of High and Low ADHD symptoms. Descriptive statistics (frequency, mean, and standard deviation) were computed to describe the frequency distribution of each variable. Chi-Square test of independence was employed to examine the association between two categorical variables. For continuous variables, independent samples t-tests were used to compare means between two groups, and One-Way Analysis of Variance (ANOVA) was employed for comparisons involving three or more groups. Following the identification of a significant overall effect for categorical variables with more than two levels (e.g., maternal education and batch year), *post-hoc* pairwise comparisons were conducted using the Bonferroni correction method to determine the specific group differences contributing to the significance. Effect sizes were reported using Cohen’s d value for the continuous variable. For the dichotomous variables, the Phi(ϕ) coefficient was used. Cramer’s V was employed to report the effect size for variables with more than two categories. All categorical variables used in the regression models, including the dichotomous outcome variable (High ADHD Symptoms = 1; Low ADHD Symptoms = 0) and binary predictor variables, were consistently coded in a binary fashion: the reference or lower category was assigned a value of 0, and the higher or target category was assigned a value of 1.

The Bi-variable logistic regression analysis at 95% confidence interval and P< 0.25, was used to identify candidate variables for multiple logistic regression analysis. The multivariable logistic regression models, at 95% CI and P< 0.05 were applied to identify the associated variables with the outcome variable.

### Ethical approval and consent to participate

The study was approved by the institutional review board of Dilla University (duirb/799/2024). All participants provided written informed consent and were informed about the study’s goals, as well as their right to withdraw at any time. Confidentiality was maintained, and data access was restricted to the principal investigator and the research advisors.

## Results

### Prevalence of high ADHD symptoms

Among all respondents, 25.1% (95% CI: 22.1%–27.7%) were categorized as having high ADHD symptoms, scored above 30 out of 72 on the ADHD assessment scale.

### Sociodemographic characteristics

This study enrolled 513 respondents who provided complete information for the current study. Out of all respondents, 218 (42%) were female. As compared to males, females were more likely to be in the High ADHD group than Low ADHD group (**Table 1**).

**Table 1:** Sociodemographic characteristics of respondents attending in Dilla university (n=513).

Characteristic	Entire sample (N = 513)	Low ADHD symptoms (n=388)	High ADHD symptoms (n=125)	F(df)	Effect size	P-value		
Age (Mean ± SD)	23.4(± 5.4)	22.2(± 4.3)	21.3(± 2.6)	7.96 (1) d= -0.22)		0.01		
Family monthly income (Mean ± SD)	10000 (2450) ETB	9556 (1769)	3467 (1043)	2211.09 (1), (d = -3.75		<0.01		
Student pocket money (Mean ± SD)	1500 (350) ETB	1295 (235)	456 (75)	3757.60 (1), d=-4.03		<0.01		
Characteristic	Entire Sample (N = 513)	Low ADHD Symptoms (n=388)	High ADHD Symptoms (n=125)	X^2^(df)	Effect size	P-value		
Gender				19.7 (1)	0.20	<0.001		
Female	218	143	75					
Male	295	245	50					
Residence				41.7 (1)	0.28	<0.001		
Rural	293	190	103					
Urban	220	198	22					
Presence stable parent				3.90 (1)	0.08	0.06		
Yes	164	133	31					
No	349	255	94					
Mother alive				0.01 (1)	0.005	0.91		
Yes	467	354	113					
No	46	34	12					
Father alive				2.03 (1)	0.06	0.20		
Yes	446	342	104					
No	67	46	21					
Father education				2.15 (3)	0.065	0.54		
Unable to read write	72	54	18					
Primary	118	84	34					
Secondary	221	169	52					
Higher (college and above)	102	81	21					
Mother education				35.6 (3)	0.26	<0.001	*Post hoc* analysis (P-value)	
Unable to read write (1)	165	100	65				(1) vs (2)	0.0135
							(1) vs (3)	< 0.0001
Primary (2)	112	84	28				(1) vs (4)	< 0.0001
Secondary (3)	124	105	19				(2) vs (3)	0.0673
Higher (college and above) (4)	112	99	13				(2) vs (4)	0.0118
							(3) vs (4)	0.4140
Family size				0.34 (1)	0.026	0.55		
≥ 5	344	257	87					
< 5	169	131	38					
Sibling order				14.1 (1)	0.17	<0.001		
< 3^rd^	144	92	52					
≥3^rd^	369	296	73					

### Academic related characteristics

This study’s findings revealed that 92 (17.9%) of them were from the college of medicine and health science, and 197 (38.4%) were 4th year students. There was a significant difference across batch years, with the 4th year students having lower ADHD symptoms compared to the other batches (**Table 2**).

**Table 2:** Academic characteristics of respondents attending at Dilla University, (n=513).

Characteristic	Entire sample (N = 513)	Low ADHD symptoms (n=388)	High ADHD symptoms (n=125)	X^2^ (df)	Effect size	P-value		
Faculty				8.25 (8)	0.12	0.84		
Engineering and Technology	32	23	9					
Computer science	36	21	15					
Business and Economics	77	60	17					
Agricultural and Natural resource	62	47	15					
Medicine and Health	92	73	19					
Natural and computational	55	41	11					
Social science and humanities	73	55	18					
Educational and behavioral science	55	38	17					
Law	31	23	8					
Batch Year				12.0 (3)	0.15	0.007	*Post hoc* analysis (P-value)	
2^nd^ Year (1)	150	118	32				(1) vs (2)	0.372
3^rd^ Year (2)	73	49	24				(1) vs (3)	1.00
							(1) vs (4)	0.14
4^th^ Year (3)	197	160	37				(2) vs (3)	0.08
5^th^ Year (4)	93	61	32				(2) vs (4)	1.00
							(3) vs (4)	0.02
Do you have difficulty answering an essay question				0.25 (1)	0.02	0.69		
Yes	159	118	41					
No	354	270	84					
GPA (current grade point average), (Mean score value =3.43)				13.3 (1)	0.16	<0.001		
< 3.43	272	188	84					
≥3.43	241	200	41					
History of academic discipline problems				4.14 (1)	0.08	0.05		
Yes	67	44	23					
No	446	344	102					
Do you finish exams before end time				1.01(=1)	0.04	0.36		
Yes	221	172	49					
No	292	216	76					
Do you properly read exam instructions before you start to answer?				0.44 (1)	0.02	0.57		
Yes	349	267	82					
No	164	121	43					
Average study hours for exam				47.4 (1)	0.30	<0.001		
< 4 hr.	221	134	87					
≥4 hr.	292	254	38					

### Psychosocial and health related characteristics

Students with High ADHD Symptoms were disproportionately represented in high social media use, test anxiety (Yes), moderate and good social support, poor meta-cognitive ability category, and having a childhood infection history (**Table 3**).

**Table 3:** Psychosocial and health related factors of respondents attending at Dilla University, (n=513).

Characteristic	Entire Sample (N = 513)	Low ADHD symptoms (n=388)	High ADHD symptoms (n=125)	X^2^(df)	Effect size	p-value	*Post hoc* analysis (P-value)	
Social support				44.5 (2)	0.29	<0.001		
Poor (3–8) (1)	354	297	57				(1) vs (2)	<0.001
Moderate (8–12) (2)	96	51	45				(1) vs (3)	0.003
Good (12–14) (3)	63	40	23				(2) vs (3)	0.25
Social media				75.8 (1)	0.38	<0.001		
High ≥17/30)	241	140	101					
Low (<17/30)	272	248	24					
Depression				3.68 (1)	0.08	0.07		
No (<13/63)	177	125	52					
Yes (≥13/63)	336	263	73					
Sleep quality				3.37 (1)	0.08	0.08		
Good	159	112	47					
Poor	354	276	78					
Test anxiety				49.6 (1)	0.31	<0.01		
No	243	218	25					
Yes	270	170	100					
Task coping				0.77 (1)	0.03	0.43		
Low task coping	235	182	53					
High task coping	278	206	72					
Emotional coping				1.02 (1)	0.04	0.36		
Low emotional coping	299	231	68					
High emotional coping	214	157	57					
Avoidance				2.69 (1)	0.07	0.12		
Low avoidance coping	140	113	27					
High avoidance coping	373	275	98					
Meta cognitive ability				41.3 (1)	0.28	<0.001		
Poor	296	193	103					
Good	217	195	22					
Perceived stress				0.96 (1)	0.04	0.40		
Yes	71	57	14					
No	442	331	111					
Self esteem				0.09 (1)	0.01	0.88		
Low	53	41	12					
High	460	347	113					
Current history of substance use				11.3 (1)	0.14	0.001		
Yes	123	77	46					
No	390	311	79					
Lifetime family history of substance use				2.16 (1)	0.06	0.18		
Yes	74	61	13					
No	439	327	112					
Family history psychiatry illness				0.06 (1)	0.01	0.79		
Yes	54	21	33					
No	459	367	92					
Child hood infection				33.01 (1)	0.254	<0.001		
Yes	171	103	68					
No	342	285	57					
History of head injury				2.27 (1)	0.06	0.13		
Yes	60	32	28					
No	453	356	97					

### Multivariate regression analysis of risk factors of high ADHD symptoms

Respondents from rural areas were 2.21 times more likely to exhibit high ADHD symptoms compared to those from urban areas. Those whose mothers were unable to read or write were 2.34 times more likely to experience high ADHD symptoms compared to those whose mothers had a college education or higher. Respondents with a history of childhood infections were 1.21 times more likely to report high ADHD symptoms than those without such a history (**Table 4**).

**Table 4:** Regression analysis results of factors associated with High ADHD among respondents attending at Dilla University, (n=513).

Variables	Category	ADHD		COR (95% CI)	P – value	AOR (95% CI)	P- value
		Low	High				
Gender	Female	143	75	1			
	male	245	50	2.57 (0.32 -3.79)	0.13	1.32(0.23-1.95)	0.12
Sibling order	< 3^rd^	92	52	0.43(0.27 -1.53)	0.14	1.03(0.57-1.23)	0.11
	>3^rd^	296	73	1			
History of head injury	Yes	32	28	0.31 (0.14 - 1.60)	0.16	0.34(0.02-1.46)	0.13
	No	356	97	1			
Residence	Rural	190	103	4.49(2.78-7.25)	0.001	2.21(1.65-3.12)	0.01
	Urban	198	22	1		1	
Mother education	Unable to read write	100	65	4.10(2.22-7.58)	0.001	2.34(1.893.23)	0.01
	Primary	84	28	2.10(1.06-4.13)	0.01	1.13(1.05-2.24)	0.13
	Secondary	105	19	1.15(0.57-2.36)	0.46	1.06(0.32-1.89)	0.65
	Higher (college and above)	99	13	1		1	
History of childhood infection	Yes	103	68	2.25 (1.42-3.55)	0.01	1.21 (1.13-2.23)	0.02
	No	285	57	1		1	
Social support	Poor	297	57	2.34(1.35 - 4.05)	0.02	1.23(0.98-2.43)	0.07
	Moderate	51	45	1.73(0.92 - 3.25)	0.085	1.13(0.75-2.45)	0.13
	Strong	40	23	1			

### Multivariate regression analysis of impairing outcomes of ADHD symptom

Compared to those with Low ADHD symptoms, those with High ADHD symptoms were 2.05 times more likely to have a GPA< 3.43. Participants who studied for less than four hours per day were 2.26 times more likely to fall under the category of high ADHD symptoms than those who studied for more than four hours. Respondents with test anxiety were 2.78 times more likely to be under the category of ADHD symptoms. Respondents with poor metacognitive ability were 2.86 times more likely to be categorized under high ADHD symptoms. Those respondents with high social media use were 3.42 times more likely to be classified under the category of ADHD symptoms (**Table 5**).

**Table 5:** Regression analysis results of factors associated with High ADHD symptom among respondents attending at Dilla University, (n=513).

Variables	Category	ADHD		COR (95% CI)	P – value	AOR (95% CI)	P- value
		Low	High				
Average study hour per day	< 4hr	134	87	3.42(2.2-5.33)	0.001	2.26 (2.14 -3.75)	0.01
	> 4hr	254	38	1		1	
Test anxiety	Yes	170	100	5.13 (3.17-8.31)	0.001	2.78(2.02-4.13)	0.02
	No	218	25	1		1	
Meta cognitive ability	Poor	193	103	4.73 (2.86-7.81)	0.001	2.86(2.17-4.67)	<0.001
	Good	195	22	1		1	
Social media	High	140	101	7.45 (4.56-12.18)	<0.001	3.42(2.21-4.63)	0.001
	Low	248	24	1			
Grade point average	< 3.43	188	84	2.05(1.36-3.09)	0.001	1.23(1.14-2.24)	0.03
	≥3.43	200	41				
Finishing exams early before end time	Yes	172	49	1			
	No	216	76	1.57(0.90 - 2.24)	0.12	1.43(0.84-1.67)	0.14
Not reading instructions	Yes	267	82	1			
	No	121	43	1.28 (0.86 - 1.89)	0.21	1.15(0.73-1.32)	0.17
Academic discipline	Yes	44	23	1.73 (0.66 - 2.82)	0.18	1.52(0.42-1.83)	0.10
	No	344	102	1			
Task Coping	High	206	72	1			
	Low	182	53	0.70 (0.44 - 1.11)	0.12	0.56(0.34-1.04)	0.16

COR, Crude odds ratio; AOR, Adjusted odds ratio; CI, Confidence interval; P-value-probability value.

## Discussion

Attention Deficit Hyperactivity Disorder (ADHD) presents a significant challenge for many students, influencing their academic performance and potentially hindering their educational progress. The results of this study highlight a clear association between the severity of ADHD symptoms and academic outcomes in a sample of undergraduate Dilla University students, Southern Ethiopia.

### Magnitude of high ADHD symptoms

In this study, 25.1% of respondents were identified with high ADHD symptoms, which is consistent with studies conducted in the USA, 25% (91), Kenya, 23.7% (15), South Africa, 25.9% (92), and Gondar university, Ethiopia, 20% (17). However, the prevalence found in this study is higher than studies done in the UK (7%) (6), China,(8% (93), New Zealand, (8.1% (94), Iran, (15.4% (95), and Kenya, (8.7% (15) or 9.2% (96). The differences in high ADHD symptoms might be attributed to variations in the ADHD screening tools used, socioeconomic status, culture, geography, and infrastructure. Notably, this study employed an 18-item ADHD screening tool, while many of the referenced studies used an 8-item scale, potentially accounting for the differences in prevalence rates. Moreover, the prevalence of high ADHD symptoms in this study was lower than the studies done in Pakistan (34.8%) (9), and Brazil (36.8%) (14). This discrepancy may be attributed to participant characteristics; for instance, participants in the Northeast United States were first-year students, and the studies in Pakistan and Brazil focused on medical students. However, the current study samples were from the College of Natural, Social, Engineering, and Health Sciences.

### Socio-demographic factors associated with ADHD symptoms

Respondents residing in rural areas were 2.21 times more likely to display high ADHD symptoms compared to their urban counterparts. This finding is in line with previous studies conducted in Germany (97) and New Zealand (98). Rural areas often face limited access to healthcare services and skilled health professionals during critical periods such as pregnancy and delivery, which may reduce risk for neurodevelopmental conditions like ADHD (99).

The cultural influences in understanding risk for elevated ADHD symptoms in rural areas might affect early screening and link to an appropriate health facility for further evaluation (100).

Additionally, socioeconomic challenges, such as poverty and limited access to mental health care, are common in rural populations and are associated with greater risk for more severe ADHD symptoms (101).

Respondents whose mothers had no formal education were 2.34 times more likely to exhibit sub-clinical threshold ADHD symptoms compared to those with more educated mothers. This is consistent with findings from Ethiopia (102), Uganda (103), Iran (104), and Oman (105). Maternal education levels significantly impact parenting practices, which in turn affect the child’s behavioral and emotional development (106, 107). Low maternal literacy is often linked to poverty, creating stressful home environments that may exacerbate of ADHD symptoms (108). Low education might contribute to reduced access to quality prenatal care, nutritional deficiencies, and heightened psychosocial stress, which are known to be correlated with ADHD symptoms (109).

### Health related factors associated with ADHD symptom

Respondents with a history of childhood infections were 1.21 times more likely to experience ADHD symptoms. This is consistent with studies conducted in Ethiopia (110), Thailand (111), and the USA (112). Infections, particularly those affecting the developing brain, can interfere with dopaminergic and neurotransmitter systems, contributing to the onset of ADHD symptoms (113, 114).

### Academic related impairing outcomes of ADHD symptom

Compared to students with Low ADHD symptoms, those with High ADHD symptoms were more likely to have a GPA less than 3.43. This finding mirrors previous studies from the USA (41) and Canada (115). Students with lower GPAs might have impairments in executive functions, such as planning, organizing, and managing time, which are critical for academic success (36, 116). ADHD symptoms, including inattention, distractibility, and impulsivity, can hinder students’ ability to complete assignments, follow instructions, and retain information, further affecting their academic performance (117–119).

Additionally, compared to students with Low ADHD symptoms, those with High ADHD symptoms were more than twice as likely to study for less than 4 hours for exams. This aligns with findings from the USA (120). Students with ADHD may struggle with attention and focus, which can lead to reduced cognitive activity, resulting in poor academic performance and low self-esteem (121, 122).

Compared to students with Low ADHD symptoms, those with High ADHD symptoms were nearly three times as likely to report experiencing High Test anxiety This finding is supported by studies from Taiwan (123), USA (124), Israel (125), and Northeast USA (126). Test anxiety, characterized by excessive worry, fear of failure, and poor concentration (127), can exacerbated by ADHD symptoms and lead to poor academic performance (128, 129).

### Psychological and cognitive function impairing outcomes with ADHD symptom

Respondents with poor cognitive function were 2.86 times more likely to be classified with ADHD symptoms. Cognitive impairments, such as difficulties with attention, working memory, focus, and impulse control, are characteristic of ADHD and can hinder students’ ability to plan, organize, and complete tasks (130, 131).

Respondents with high social media use were 3.42 times more likely to be under the category of high ADHD symptoms. This is consistent with studies from India (132), and the Netherlands (133). Social media use is a prominent procrastination mechanism for an individual with difficulties in sustained attention, executive function (EF), and initiation of demanding academic tasks. Inattentiveness, diminished concentration, and impulse control symptoms of ADHD might need regular stimulation and dopamine reward system activation, which can be achieved by social media engagement, particularly on entertainment videos, chats, and group networking. This maladaptive coping mechanism further contributes to high engagement in avoidance behavior and procrastination, and negatively impacts study duration and academic performance (134) (135).

### Study implications

The findings have significant implications for higher education institutions, public health policy, and clinical practice in Ethiopia, suggesting a need for early identification and intervention through implementing ADHD symptom screening during student intake. Institutions should design comprehensive intervention programs that include psychosocial and mental health services, regular follow-ups, and continuous personal development training to mitigate the impact of ADHD symptoms on students’ personal, social, and academic lives. Furthermore, they should provide academic support activities like tutoring, study skills workshops, and extended exam time, and prepare a conducive and inclusive learning environment that minimizes distractions. For social development organizations, the findings imply a need to increase access to healthcare and quality education in rural areas, offer support programs for students from low-income families (such as scholarships and mentorship), and provide educational programs for parents and caregivers on childhood healthcare, neurodevelopmental conditions, and mental health. Clinically, the study underscores the necessity of screening for ADHD symptoms during routine childhood pediatric visits and highlights the influence of childhood infections on neurocognitive development.

### Recommendations for future research

To build upon these findings, further research is needed across different study settings. First, studies should aim to examine the direct relationship between the specific underlying mechanisms of each associated factor and the manifestation of ADHD symptoms among students in higher education. Second, the study findings would benefit from work focused on the design and evaluation of targeted interventions specifically aimed at improving academic outcomes for students presenting with ADHD symptoms. Furthermore, longitudinal studies would be helpful to investigate the long-term impact of ADHD symptoms on students’ overall educational attainment and subsequent career trajectories, and other important areas of life.

### Limitations

The cross-sectional nature of the study limits the ability to reflect the causal relationship between associated factors and ADHD symptoms. ADHD symptoms were assessed using self-report measures, which may be subject to response bias. The key limitation is the definition of family history, which relies on student reports of any parental substance use. This broad, non-diagnostic measure does not confirm a substance use disorder (SUD). Future studies should use diagnostic tools or validated SUD scales to confirm the specificity of these findings. This study lacked a clinical examination to confirm ADHD diagnoses, and a full symptom assessment using a checklist was not conducted. As a result, the prevalence of ADHD symptoms in this study may be overestimated, as is common with screening instruments. The study was done on college students, which might hinder generalizing the findings to other non-clinical communities. The study used some study variables with single-item questions, such as stable parent, which might not reflect the quality of the parental relationship. Furthermore, the non-clinical sample limits the generalizability of these findings to diagnosed clinical populations. Future longitudinal studies are needed to determine the causal relationships and to disentangle the complex interplay between ADHD symptoms and associated sociodemographic, psychosocial, and cognitive factors.

## Conclusions

This study reveals a significant prevalence of high ADHD symptoms among higher education students in Southern Ethiopia, highlighting the importance of addressing this neurodevelopmental condition within this population. Notably, respondents from rural areas, those with mothers who were unable to read or write, and a history of childhood infections, underscore the influence of socio-demographic and health-related factors on the manifestation of these symptoms. The study findings also revealed the impairing outcome of ADHD symptoms on academic score, cognitive function, exam-related anxiety, study hours, and social media use.

Therefore, interventions and support systems tailored to address these multifaceted challenges are crucial for improving the academic success and quality of life of university students in Southern Ethiopia. Early screening and detection of ADHD symptoms are crucial, as well as providing educational opportunities for mothers, psychosocial support to alleviate test anxiety, and strengthening social support systems to improve academic performance.

## Data Availability

The original contributions presented in the study are included in the article/**Supplementary Material**. Further inquiries can be directed to the corresponding author.
