# Supplementary material for: Prevalence and associated factors of ADHD symptoms among higher education students in Southern Ethiopia
Source: Front Psychiatry. 2026 Jan 14;16:1566847. doi: 10.3389/fpsyt.2025.1566847 (PMC12847265; doi:10.3389/fpsyt.2025.1566847)
Supplement: Supplementary file 1 [file Table1.docx]

| Characteristic | B | S.E. | Wald χ2 | p-value | Odds Ratio (OR) | 95% Confidence Interval for OR |
| --- | --- | --- | --- | --- | --- | --- |
| Continuous Variables |  |  |  |  |  | Lower – Upper |
| Age (per year increase) | 0.09 | 0.03 | 9.00 | 0.37 | 0.91 | 0.85 - 1.97 |
| Family monthly income (per 1000 ETB) | 0.15 | 0.04 | 14.06 | 0.31 | 0.56 | 0.40 -1.93 |
| Student pocket money (per 100 ETB) | 0.22 | 0.05 | 19.36 | 0.28 | 0.80 | 0.73 - 1.88 |
| Categorical Variables (Reference Group) |  |  |  |  |  |  |
| Gender (Ref: Female) |  |  |  |  |  |  |
| Male | 0.69 | 0.23 | 9.00 | 0.131 | 1.50 | 0.32 -2.79 |
| Residence (Ref: Urban) |  |  |  |  |  |  |
| Rural | 1.10 | 0.24 | 20.94 | 0.001 | 4.49 | 2.87 - 7.81 |
| Mother education (Ref: Higher) |  |  |  |  |  |  |
| Unable to read/write | 1.50 | 0.35 | 18.37 | 0.001 | 4.10 | 2.22 - 7.58 |
| Primary | 0.85 | 0.38 | 5.02 | 0.025 | 2.10 | 1.06 - 4.13 |
| Secondary | 0.40 | 0.35 | 1.31 | 0.252 | 1.15 | 0.57- 2.36 |
| Sibling order (Ref: >3rd |  |  |  |  |  |  |
| < 3^rd^ | 0.75 | 0.26 | 8.30 | 0.14 | 1.12 | 0.97 -1.53 |

| Academic Performance |  |  |  | p-value |  | Lower - Upper |
| --- | --- | --- | --- | --- | --- | --- |
| GPA (Ref: > 3.43) |  |  |  |  |  |  |
| < 3.43 | 0.78 | 0.20 | 15.21 | 0.001 | 2.05 | 1.36 - 3.09 |
| Study Habits |  |  |  |  |  |  |
| Avg. study hours for exam (Ref: > 4 hr) |  |  |  |  |  |  |
| < 4 hr | 1.43 | 0.21 | 46.40 | 0.001 | 3.42 | 2.2 – 5.33 |
| Do you finish exams before end time (Ref: Yes) |  |  |  |  |  |  |
| No | 0.45 | 0.18 | 6.25 | 0.12 | 1.57 | 0.90 - 2.24 |
| Reading exam instructions (Ref: Yes) |  |  |  |  |  |  |
| No | 0.25 | 0.20 | 1.56 | 0.212 | 1.28 | 0.86 - 1.89 |
| Academic Discipline (Ref: No) |  |  |  |  |  |  |
| History of discipline problems | 0.55 | 0.25 | 4.84 | 0.18 | 1.73 | 0.66 - 2.82 |
| Essay Questions (Ref: No difficulty) |  |  |  |  |  |  |
| Difficulty answering essay question (Yes) | 0.38 | 0.20 | 3.61 | 0.357 | 1.46 | 0.99 - 2.15 |
| Batch Year (Ref: 2nd Year) |  |  |  |  |  |  |
| 3rd Year | 0.52 | 0.32 | 2.64 | 0.259 | 1.68 | 0.90 - 3.14 |
| 4th Year | 0.10 | 0.26 | 0.15 | 0.698 | 0.90 | 0.53 - 1.52 |
| 5th Year | 0.61 | 0.30 | 4.11 | 0.323 | 0.99 | 0.92 - 3.33 |
| Faculty (Ref: Medicine and Health) |  |  |  |  |  |  |
| Engineering and Technology | 0.55 | 0.51 | 1.16 | 0.281 | 0.58 | 0.21 - 1.58 |
| Computer science | 0.40 | 0.42 | 0.90 | 0.343 | 1.49 | 0.66 - 3.37 |
| Business and Economics | 0.62 | 0.36 | 2.97 | 0.285 | 0.54 | 0.27 - 1.09 |
| Agricultural and Natural resource | 0.09 | 0.39 | 0.05 | 0.823 | 0.91 | 0.42 - 1.98 |
| Natural and computational | 0.35 | 0.41 | 0.73 | 0.393 | 0.70 | 0.32 - 1.54 |
| Social science and humanities | 0.30 | 0.38 | 0.63 | 0.427 | 0.74 | 0.35 - 1.56 |
| Educational and behavioral science | 0.01 | 0.38 | 0.00 | 0.999 | 1.01 | 0.48 - 2.12 |
| Law | 0.80 | 0.56 | 2.04 | 0.353 | 0.45 | 0.15 - 1.34 |
| Psychosocial Factors |  |  |  |  |  | Lower - Upper |
| Social Support (Ref: Good) |  |  |  |  |  |  |
| Poor (3-8) | 0.85 | 0.28 | 9.26 | 0.02 | 2.34 | 1.35 - 4.05 |
| Moderate (8-12) | 0.55 | 0.32 | 2.96 | 0.085 | 1.73 | 0.92 - 3.25 |
| High Social Media Use (Ref: Low) |  |  |  |  |  |  |
| Yes ≥17/30 | 1.85 | 0.20 | 85.56 | 0.001 | 3.42 | 2.21- 4.63 |
| Depression (Ref: No) |  |  |  |  |  |  |
| Yes (≥13/63) | 0.40 | 0.23 | 3.01 | 0.283 | 1.49 | 0.95 - 2.33 |
| Sleep Quality (Ref: Good) |  |  |  |  |  |  |
| Poor | 0.50 | 0.22 | 5.17 | 0.29 | 0.65 | 0.08 - 1.53 |
| Test Anxiety (Ref: No) |  |  |  |  |  |  |
| Yes | 1.20 | 0.20 | 36.00 | 0.001 | 5.13 | 3.17- 8.31 |
| Coping Strategies (Ref: High) |  |  |  |  |  |  |
| Low Task Coping | 0.35 | 0.23 | 2.32 | 0.128 | 0.70 | 0.44 - 1.11 |
| Low Emotional Coping | 0.18 | 0.23 | 0.61 | 0.435 | 0.84 | 0.53 - 1.33 |
| High Avoidance Coping | 1.05 | 0.27 | 15.00 | 0.26 | 0.84 | 0.69 - 1.84 |
| Meta-Cognitive Ability (Ref: Good) |  |  |  |  |  |  |
| Poor | 1.60 | 0.21 | 57.97 | 0.001 | 4.73 | 2.86- 7.81 |
| Self-Esteem (Ref: High) |  |  |  |  |  |  |
| Low | 0.30 | 0.38 | 0.62 | 0.431 | 1.35 | 0.64 - 2.83 |
| Perceived Stress (Ref: No) |  |  |  |  |  |  |
| Yes | 0.90 | 0.35 | 6.61 | 0.30 | 0.41 | 0.20 - 1.84 |
| Clinical/Family History |  |  |  |  |  |  |
| Current Substance Use (Ref: No) |  |  |  |  |  |  |
| Yes | 0.75 | 0.24 | 9.77 | 0.28 | 0.52 | 0.32 - 1.41 |
| Family History Psychiatry Illness (Ref: No) |  |  |  |  |  |  |
| Yes | 1.90 | 0.35 | 29.40 | 0.290 | 0.86 | 0.34 - 1.36 |
| History of Head Injury (Ref: No) |  |  |  |  |  |  |
| Yes | 1.30 | 0.30 | 18.78 | 0.17 | 0.67 | 0.04 - 1.60 |
| Childhood Infection (Ref: No) |  |  |  |  |  |  |
| Yes | 0.65 | 0.22 | 8.77 | 0.003 | 2.25 | 1.42- 3.55 |
